# Mindfulness in pain self-control of people with chronic pain: a cross-sectional study

**DOI:** 10.1192/j.eurpsy.2022.1700

**Published:** 2022-09-01

**Authors:** C. Laranjeira, A. Querido

**Affiliations:** Polytechnic of Leiria, School Of Health Sciences/ Citechcare, Leiria, Portugal

**Keywords:** Mindfulness, self-control, knowledge, chronic pain

## Abstract

**Introduction:**

Chronic pain has a significant impact on individuals’ daily lives, and its control is essential for improving quality of life. In this sense, the practice of Mindfulness is a useful non-pharmacological technique for self-management of chronic pain.

**Objectives:**

This study aims to identify the level of knowledge regarding the effectiveness of Mindfulness for self-control of pain by people with chronic pain.

**Methods:**

A cross-sectional study was conducted on a sample of 23 adults with chronic pain. The online survey assessed sociodemographic characterization, Brief Pain Inventory, and knowledge/opinion about the effectiveness of mindfulness strategies.

**Results:**

The sample consisted mostly of middle-aged women, with family support, employed and with higher academic qualifications. 47.8% of the sample had experienced pain for over 20 years with the most prevalent diagnosis being fibromyalgia. The average intensity of chronic pain corresponded to moderate pain and the level of acceptance of it was low, interfering in instrumental activities of daily life. Although they had never tried the technique, most of the sample knew what mindfulness consisted of, considering it as a viable option for self-management of chronic pain. Moderate and positive correlations were found between the level of acceptance of pain and greater availability for the practice of mindfulness (rho=.137; p<.001), the same happened between satisfaction with the practice of mindfulness and self-control of pain (rho=.259; p<.001).

**Conclusions:**

Our findings outline the need to include non-pharmacological measures such as mindfulness in therapeutic schemes for chronic pain management, given the manifest interest of this population.

**Disclosure:**

No significant relationships.

